# High-level activity and access to sport following lower limb amputation due to malignant musculoskeletal tumors versus trauma: a prospective comparative study

**DOI:** 10.1007/s00402-025-06037-x

**Published:** 2025-08-21

**Authors:** Astrid Schenker, Sebastian I. Wolf, Merkur Alimusaj, Cornelia Putz, Burkhard Lehner, Julia Block

**Affiliations:** 1 Heidelberg University Hospital, Heidelberg, Germany; 2 University of Heidelberg, Heidelberg, Germany

**Keywords:** Amputation, High-level activity, Tumor amputations, Sports, Outcome measurements, Rehabilitation, CHAMP

## Abstract

**Introduction:**

Patients with musculoskeletal tumors who undergo amputation show reduced physical function. To give these patients better expectations of their future physical capabilities and to enhance the evaluation and development of postoperative rehabilitation, we investigated high-level activity and access to sport using objective measurements and questionnaires. We then compared the results to those of amputees who had undergone amputation due to trauma.

**Materials and methods:**

In a prospective, monocentric study, we evaluated and correlated the results of the conventional mobility tests such as “timed-up-and-go” test (TUG), “2-Minute-Walk-Test” (2MWT), as well as the “10-Meter-Walk-Test” (10MWT) with a test for high-level activity. In this contribution, the Comprehensive High-Level Activity Mobility Predictor (CHAMP), originally developed for male servicemembers, was tested for feasibility in our cohort. We examined eleven patients who underwent amputation due to primary malignant bone or soft-tissue sarcomas and compared the results with ten patients who experienced traumatic amputation, along with seventeen patients in the healthy control group.

**Results:**

Patients with lower leg amputations due to malignant musculoskeletal tumors exhibited superior outcomes in general mobility and high-level activity mobility compared to those with traumatic amputations. Using a questionnaire, we were able to demonstrate that patients suffering after amputation because of musculoskeletal tumor exhibited higher motivation and a greater sense of health and well-being compared to participants who had undergone traumatic amputation.

**Conclusions:**

The CHAMP can be utilized as a complementary tool in the rehabilitation of amputees to objectively assess high-level mobility and to guide targeted training and therapeutic interventions.

**Supplementary Information:**

The online version contains supplementary material available at 10.1007/s00402-025-06037-x.

## Introduction

The World Health Organization’s (WHO) current recommendation for physical activity in adults is at least 150–300 min of moderate-intensity physical activity or 75–150 min of vigorous-intensity physical activity per week [1]. These recommendations also apply for disabled people and studies show that amputees in particular benefit from physical activity [2]. But despite the known benefits, studies have shown that amputation very often leads to low physical function and that only a minority of amputees engage in regular sports or physical exercise [3–6]. Factors such as pain, embarrassment, insufficient training and lack of organized sports programs for the disabled and inadequate prosthetic technology contribute to this gap [4]. Efforts to encourage and facilitate higher levels of physical activity in this population are crucial to addressing these disparities and improving their health outcomes. The primary objective following lower limb amputation is the restoration of walking ability. But knowing about the positive impact of high-level physical activity, particularly sports, on physical and mental well-being, physical fitness should be developed in amputees to the extend, that it will counteract the effects of a sedentary lifestyle on general health. Subsequently, encouraging participation in sports, either recreationally or competitively, should be promoted as a personal goal. Bragaru et al. conducted a review of several studies on amputee outcomes, concluding that sports should be integrated into rehabilitation programs [7]. Another study demonstrated that a training regimen focused on enhancing hip strength led to significant improvements in prosthetic walkers, enabling them to engage in running [8]. Amputations are relatively common among veterans who have served and have a great impact on their professional careers. Therefore, Gailey et al. developed and assessed the reliability of a high-level activity outcome measure in male servicemembers with traumatic lower-limb loss called the Comprehensive High-Level Activity Mobility Predictor (CHAMP) [9]. The CHAMP assesses coordination, power, speed, and agility in multiple planes without hopping or jumping. They demonstrated that the CHAMP can be performed safely in a clinical setting after a moderate level of mobility is achieved. Due to the importance of high-level mobility in patients following lower limb amputation, we decided to use the CHAMP as a measurement tool for our patient cohort.

Our study hypothesized that patients with tumor-related amputations would be less active and engage in fewer sports compared to those with amputations due to other causes. This hypothesis was based on the assumption that tumor patients might face delayed access to rehabilitation and carry the additional burden of cancer, potentially limiting their physical activity. To explore this, we included a control group of healthy adults with similar characteristics and a comparison group of patients with traumatic amputations, as they appear to be most comparable to the tumor group in terms of demographic and clinical features. The aim of the study was to evaluate physical activity levels, general mobility, and high-level functional performance in these groups, with the goal of informing future rehabilitation strategies and motivational approaches for patients undergoing amputation.

## Materials and methods

### Study design

**Fig. 1:**
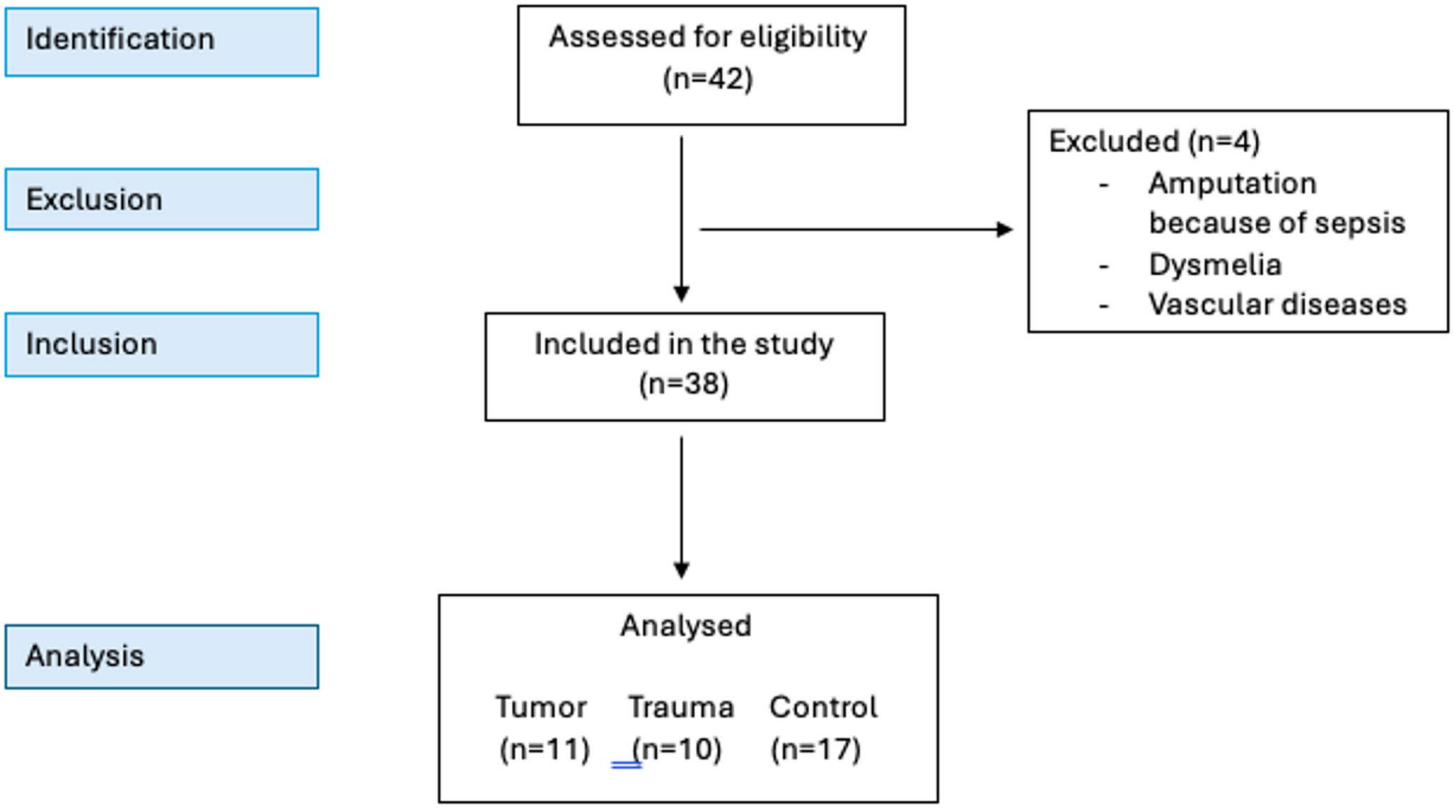
Flowchart of the participants included in the analysis

This monocentric, prospective study reports the physical activity for ambulant adults who had received lower leg amputation after tumor or trauma disease. The study received approval from the Ethic Committee (Fig. 1).

### Participants

We included 11 patients who received lower limp amputation (LLA) following tumor surgery and compared them to a group of ten patients receiving LLA due to trauma. 17 healthy adults served as control group. Participants were recruited through three main channels like our weekly outpatient clinic, where they were informed about the study and invited to participate. Some participants were recruited via an inhouse prosthetic workshop, where certified prosthetists identified suitable candidates and facilitated their enrollment in the study. Moreover, participants were recruited through “Anpfiff ins Leben,” a registered association that promotes sports among individuals following amputation and other disabilities. This organization played a crucial role in reaching out to potential participants and encouraging their involvement. Inclusion criteria comprised age ≥ 16 years, the ability to read and write, and the presence of a unilateral or bilateral lower limb amputation. To minimize the potential influence of treatment-related side effects—particularly those associated with adjuvant chemotherapy—a minimum interval of two years between completion of tumor therapy and study enrollment was required. Exclusion criteria was inability to fill in questionnaires even with support or loss of balance due to vestibular, cerebral or visual impairment. Additionally, we excluded other causes for amputation such as sepsis, vascular disease and dysmelia. After recruitment of amputees we recruited the control group which were mostly physician collegues and aimed for a reasonable degree of similarity between the controls and the study groups concerning age, sex, height and weight.

### Assessment

#### General activity

To obtain information on general mobility, activities of daily life, prosthetics and general health, we used standardized validated questionnaires in combination with functional tests integrated in our AMP registry (patient registry after limb loss), filled in by patients, physicians and specialized prosthetists [10]. Physical activity is recorded using the Locomotor Capabilities Index (LCI) and the LCI-HDS (LCI-Heidelberg Score). The LCI is a 4-level ordinal scale self-report measure that addresses capacity issues of transferring, walking and climbing stairs and has been validated for people with amputation of lower limb [11, 12]. The LCI evaluates various activities with a prosthesis whether they cannot be carried out (0 points) or can be carried out freely without assistants (4 points). The activities surveyed are divided into seven “Basic Activities” (BAS) and seven “Advanced Activities” (AAS) with a maximum achievable score of 56 points [13]. The LCI was additionally supplemented with 4 questions (LCI-HD), which assess the one-legged stance on the affected side, donning and doffing the prosthesis, getting in and out of a car, and descending stairs alternately, with equal scoring (0–4 points) and a maximum score of 16 points. In order to objectify general physical activity, we asked about the estimated walking distance and used functional tests such as the “timed-up-and-go” test (TUG) [14], “2-Minute-Walk-Test” (2MWT) [15], as well as the “10-Meter-Walk-Test” (10MWT) [16]. All tests were performed with the current prosthesis and additional assistive devices like crutches if participants used these in their everyday life. In some subjects some of the tests were performed with and without prosthesis. The TUG test begins with rising from a seated position, followed by walking three meters, performing a 180° turn, and then walking back to sit down again. The time taken to complete this sequence provides an indication of general mobility. The 2MWT measures the distance walked in two minutes, representing endurance and the ability to maintain continuous walking. Along with the 2MWT, the 10MWT is also conducted to assess current walking speed [16]. Data collection took place at an indoor gymnasium hall. A physical therapist as well as a researcher of the motoric skills laboratory, experienced in performance-based outcome measures, guided the process.

#### High-level-activity

**Fig. 2:**
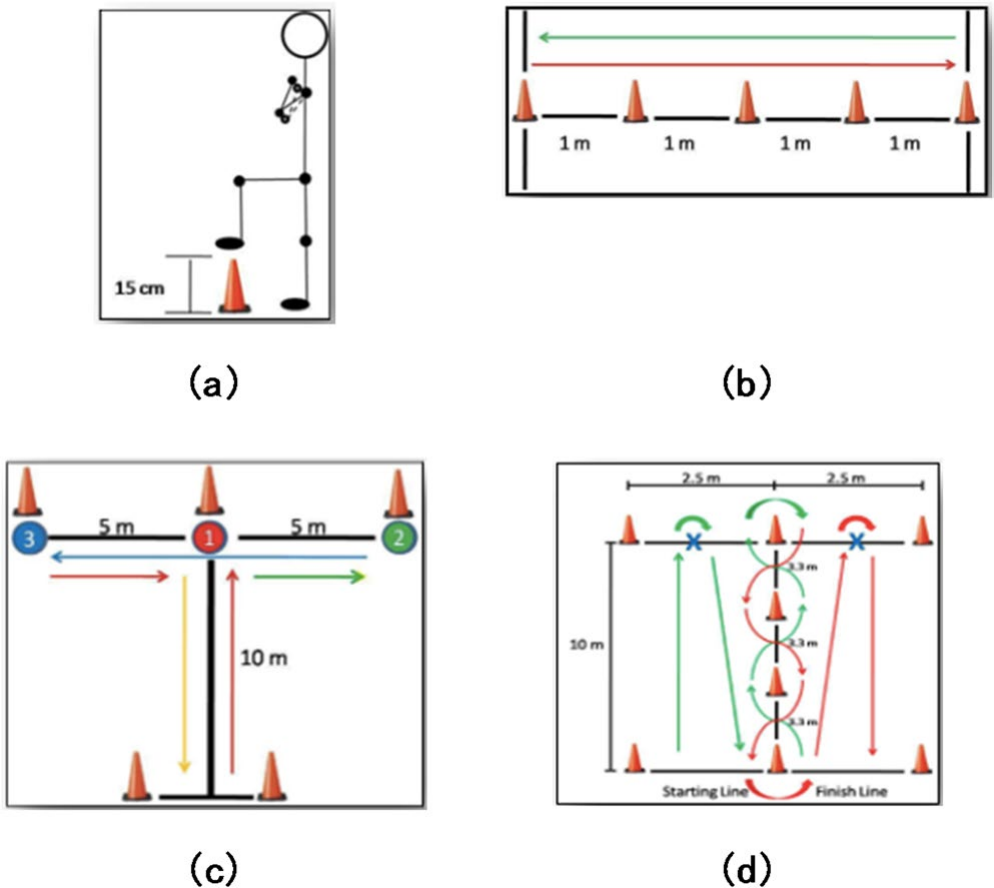
CHAMP – Comprehensive High-Level Activity Mobility Predictor: This includes four separate tests, which are evaluated using a table ranging from 0 to 10 points. With the four tests, the “Single Limb Stance (SLS)” (**a**), modified “Edgren Side Step Test” (ESST) (**b**), the “T-Test” (**c**), and the “Illinois Agility Test” (IAT) (**d**), sport-specific movements are assessed

In order to be able to better assess the athletic ability of very mobile test subjects, we expanded these functional tests, which were already collected as part of the AMP registry, to include the Comprehensive High-Level Activity Mobility Predictor (CHAMP) [9, 17]. This includes four separate tests, which are evaluated using a table with 0–10 points. The four tests, the “Single Limb Stance (SLS)”, modified Edgren Side Step Test (ESST), the T-Test and the Illinois Agility Test (IAT) are used to test sport-specific movements (Fig. 2).

CHAMP was administered and scored using the developers’ published instructions [17]. For consistency, there were the same two administrators who knew about all the test instructions and demonstrated each CHAMP task to ensure the participant understood the tasks prior to administration. No verbal motivation or performance enhancement suggestions were provided during the CHAMP instruction or test administration. Each CHAMP task was scored individually, and a composite score (ranging from 0 to 40) was calculated. Participants wore their preferred walking or running prosthesis for both tests. Participants could even choose to do the tests with crutches. Tests were performed in ascending order according to difficulty starting with TUG, 2MWT, 10MWT and ending with the CHAMP. As a tool to measure an individual’s level of exertion during physical activity and for comparative analysis we used the BORG scale (ranging from 0 to 10 points) [18]. At day of data collection every assessment was demonstrated to individuals beforehand and the individuals were asked if they felt confident to perform. At any point of the protocol participants could refuse to perform an assessment.

#### Access to sports

We developed our own questionnaire to inquire about sports participation, motivations, and obstacles, modified based on the work of Jaarsma et al. [19, 20] (S1).

### Statistical methods

Descriptive statistics were used to characterize the study sample. To compare the two groups of amputees, we used the t-test for independent samples to determine significance. To examine relationships between tests within one group of amputees, we used the Pearson correlation coefficient for significance determination. Scatter plots were used to visualize the correlations. For all tests, the significance level was set at *p* < 0.05. A p-value less than 0.05 was considered statistically significant. All statistical analyses were conducted using Excel worksheets (Microsoft; Redmond, Washington).

## Results

### Patient characteristics

Our cohort comprised eleven amputees due to musculoskeletal tumor, including four females and seven males, with a mean age of 24.6 years at the time of amputation, a mean age of 36.5 years at the time of assessment, and consequently a mean of 12.3 years between amputation and assessment. The group exhibited a diverse range of surgical interventions: four underwent transfemoral amputation, three received transtibial amputation, two underwent hemipelvectomy and two underwent rotationplasty, categorized under “others” (Table 1).

**Table 1:** Patient characteristics

Reason for amputation	Tumor	Trauma	Control
Total	11	10	17
Female	4	3	9
Male	7	7	8
Mean age at the time of amputation (min-max)	24.6 (9–49)	33.8 (4–57)	-
Mean age at the time of assessment (min-max)	36.4 (22–54)	56.1 (46–65)	37 (22–60)
Time between amputation and assessment, mean time in years (min-max)	12.3 (2–35)	20.2 (2–42)	-
Level of amputation			
TT^1^	3	4	-
TF/KX^2^	4	6	-
HP/HX^3^	2	-	-
Others	2		

^1^TT = transtibial

^2^TF/KX = transfemoral/knee-disarticulation

^3^HP/HX = hemipelvectomy/hip disarticulation

The comparative trauma group consisted of 10 participants, divided into seven males and three females. The age at the time of surgery ranged from 4 to 57 years (mean 33.8 years), with a mean age of 56.1 years at the time of assessment and a mean of 20.2 years since amputation. Among them, four received transtibial amputation, and six transfemoral amputations. The control group comprised 17 healthy individuals, with a mean age of 37 years consisting of nine females and eight males. Not all patients participated in rehabilitation or physiotherapy after amputation. Serious secondary diagnoses influencing activity in the tumor group included arthrosis, nerve lesion, missing thigh musculature after tumor resection, and left ventricular insufficiency in the tumor group. Trauma group faced serious secondary diagnosis like chronic obstructive pulmonary disease (COPD), rheumatism and muscle weakness of the thigh.

### General activity

The estimated walking distance was reported by 9 of the 11 tumor patients, with a mean of 1461 m (range: 50 to 3000 m). In the trauma patient group, 7 out of 10 provided this information, with a mean walking distance of 1300 m (range: 200 to 3000 m). Table 2 summarizes the results of general mobility assessments including LCI, LCI-HD, TUG, 2MWT, and 10MWT across all groups. Tumor amputees demonstrated slightly better average performance across most tests compared to trauma amputees, though these differences were not statistically significant except for the CHAMP (*p* = 0.03).

**Table 2:** Mean for LCI, LCI-HD, and the functional tests within each group

Test	Tumor	Trauma	Control	Maximum
Mean LCI^1^	52.3 ± 6.9	49.4 ± 8.3	-	56
Mean LCI-HD^2^	15.2 ± 1.6	13.6 ± 2	-	16
Mean TUG^3^	8.8 ± 2.7	10.9 ± 5	5 ± 0.7	-
Mean 2MWT^4^ in m	166.7 ± 44.5	135.1 ± 31.3	235 ± 22	^-^
Mean 10MWT^5^ in s	7.9 ± 2.7	8.8 ± 2.5	5 ± 0.5	^-^

^1^LCI = Locomotor Capabilities Index

^2^LCI-HD = Locomotor Capabilities Index – Heidelberg Score

^3^TUG = Timed-up-and-go test

^4^2MWT = 2-Minute-Walk-Test

^5^10MWT = 10-Meter-Walk-Test”

As shown in Table 3, functional test performance varied with the level of amputation. Tumor amputees generally outperformed trauma amputees. Statistically significant differences were observed in 2MWT for the transtibial group (*p* = 0.01) and in 10MWT, where trauma patients performed better (*p* < 0.001).

**Table 3:** Mean for TUG, 2MWT and 10MWT in dependence of amputation level

	Mean TUG^4^ in s		Mean 2MWT^5^ in m		Mean 10MWT^6^ in s	
	Tumor	Trauma	Tumor	Trauma	Tumor	Trauma
TT^1^ (Prosthesis)	8.6	9.4	144.3	129.5	9.8	8.6
TF/KX^2^ (Prosthesis)	7.9	12.4	180.3	129.2	6.9	9.5
HX/HP^3^ (Prosthesis)	13.6	-	149	-	8.9	-
HX/HP (crutches)	6.6	-	226	-	5.8	-
Others	8.4	-	131.5	-	8	-

^1^TT = transtibial

^2^TF/KX = transfemoral/knee-disarticulation

^3^HP/HX = hemipelvectomy/hip disarticulation

^4^TUG = Timed-up-and-go test

^5^2MWT = 2-Minute-Walk-Test

^6^10MWT = 10-Meter-Walk-Test”

### High-level activity (CHAMP)

**Fig. 3:**
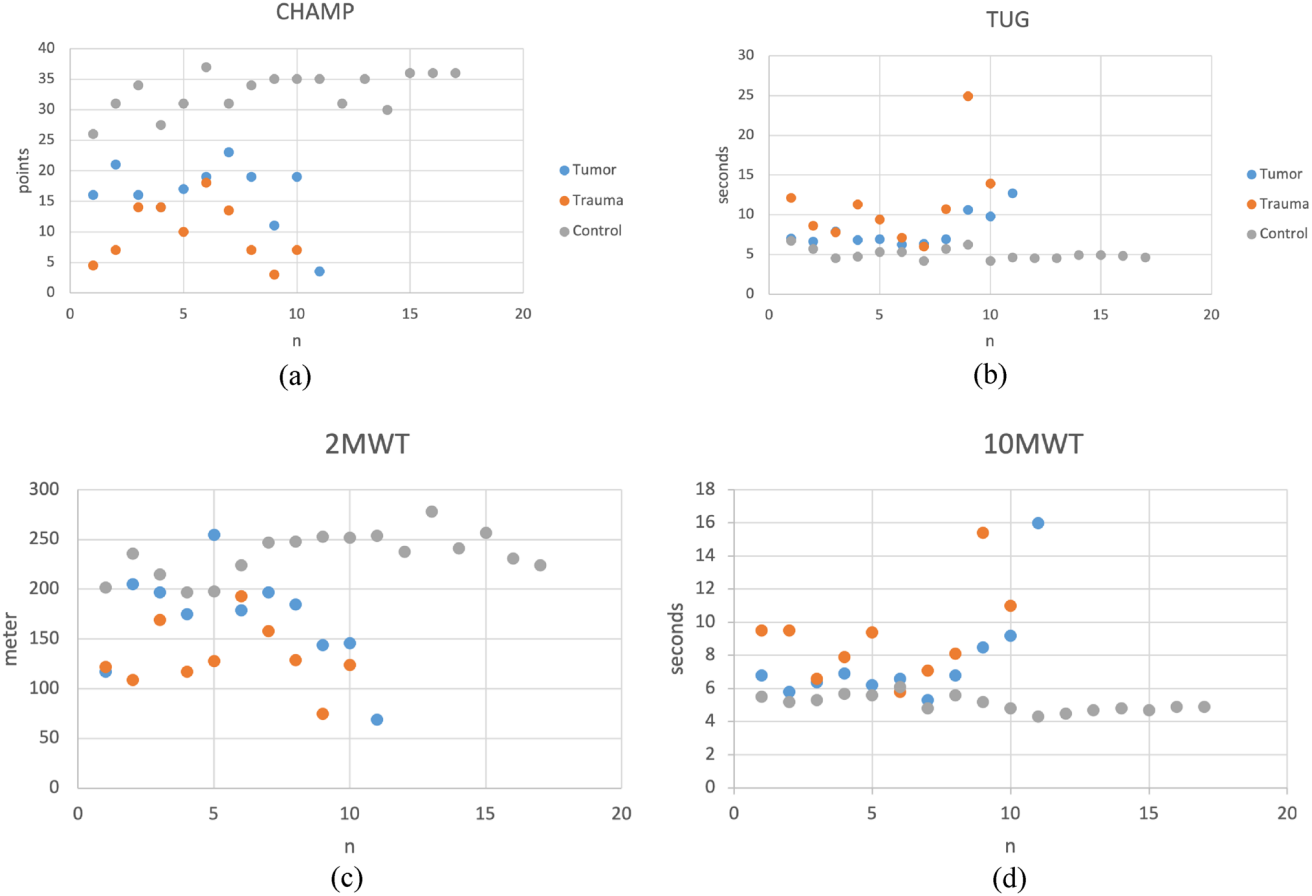
Correlation of the CHAMP to the functional tests **a** comprehensive High-Level Activity Mobility Predictor (CHAMP) in the tumor group, trauma group, and control group; **b** TUG in the three groups; **c** 2MWT in the three groups; **d** 10MWT in the three groups

CHAMP was performed by all participants with prosthesis and were repeated by few with crutches. CHAMP scores were highest in the control group, followed by tumor amputees, and lowest in trauma amputees (Fig. 3a).

**Fig. 4:**
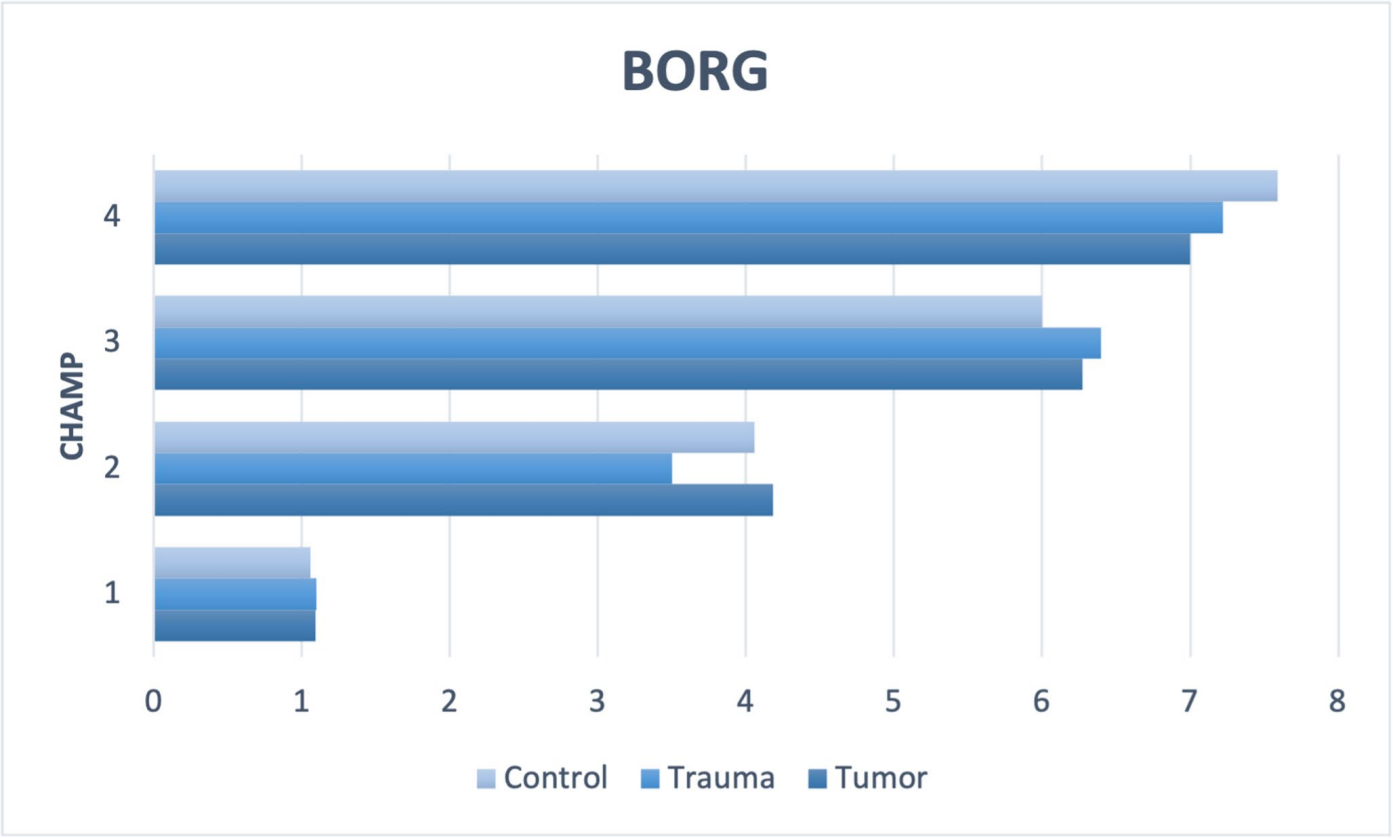
BORG scale was used to evaluate the effort of the four CHAMP items in each group with prosthesis. 1 = Single Limb Stance (SLS), 2 = modified Edgren Side Step Test (ESST), 3 = T-Test and 4 = Illinois Agility Test (IAT)

Using the Borg Scale, which was queried after each exercise, it was observed that the amputees did not perceive the individual exercises as significantly more strenuous than the control group (Fig. 4).

The CHAMP score in the tumor group demonstrated a strong positive correlation with the functional tests included in our assessment, such as TUG (*r* = 0.8, *p* < 0.001), the 2MWT (*r* = 0.8, *p* < 0,001), and the 10MWT (*r* = 0.8, *p* < 0,001). Similarly, the trauma group exhibited strong positive correlations between the CHAMP score and the functional tests: TUG (*r* = 0.7, *p* < 0.001), 2MWT (*r* = 0.8, *p* < 0,001), and 10MWT (*r* = 0.8, *p* < 0,001). These results indicate that high CHAMP scores are associated with shorter times for the TUG test, greater distance ambulated in 2 min, and shorter times needed to complete 10 m, demonstrating the score’s robust relationship with improved functional performance in both tumor and trauma groups. The ceiling effect should be considered. Age was not significantly correlated with the CHAMP score for tumor group, but it was significantly correlated for the trauma group (*r* = 0,9, *p* < 0,001). Also time since amputation correlates significantly with the CHAMP in the tumor group (*p* = 0,02) and was even highly significant for the trauma group (*p* < 0,001) (Table 4).

**Table 4:** Mean values for CHAMP in dependence of amputation level

	Mean CHAMP	
	Tumor	Trauma
TT^1^ (Prosthesis)	13.8	8
TF/KX^2^ (Prosthesis)	15.5	10.5
HX/HP^3^ (Prosthesis)	10.5	-
HX/HP (crutches)	20	-
Others	17.5	

^1^TT = transtibial

^2^TF/KX = transfemoral/knee-disarticulation

^3^HP/HX = hemipelvectomy/hip disarticulation

### Access to sports

Sports participation was more common among tumor amputees than trauma amputees. Ten out of eleven participants of the tumor group mentioned participating in some kind of sports like sitting volleyball, fitness, mountain biking, road cycling, climbing, skiing and swimming. In contrast, only four out of ten patients in the trauma group mentioned participating in sports, such as Nordic walking, aerobics, running, swimming, sitting volleyball, and leg press. Sports participation was more common among tumor amputees than trauma amputees. Detailed motivations and perceived barriers are listed in Table 5.

**Table 5:** Motivation and Obstacles for sports participation in individuals with lower limb amputations (LLA)

		Tumor *n*=	Trauma *n*=
Motivation Strong – very strong	It is good for my health	8	2
	I follow the advice of the doctors	3	1
	It helps for better mobility in daily life	6	1
	Improves experience with my prosthesis	2	1
	To shape my body	5	1
	Because of social contacts	3	1
	I want to test my limits	5	1
	It gives me a good feeling	5	3
	I get support from family and friends	2	1
	Helps me to relax/sleep better	5	1
	I get more self confidence	-	1
Obstacles Little - strong	I don’t trust myself	2	-
	Lack of offers	-	2
	No time	2	1
	Pain elsewhere	-	1
	Pain stump	1	1
	Prosthesis doesn’t fit	-	1
	Exhausting	1	-
	Costs	-	1

Modified based on the work of Jaarsma et al. [19, 20]

Gradations of answers: “no – little – strong – very strong”, n = number of answers given

## Discussion

Experimental studies have identified the effectiveness of exercise-based rehabilitation for adults after amputation on various outcomes [21, 22]. High-level rehabilitation of amputees, with opportunities to participate in physical exercise or sports, should be encouraged as soon as they are able to walk fluently. Data show that there are various advantages, such as improved role, social functioning and cost-effectiveness for cancer survivors engaging in high-level-activity compared to low-to-moderate intensity exercise [23].

Our study hypothesized that patients with tumor-related amputations would be less active and engage in fewer sports compared to those with amputations due to other causes. This hypothesis was based on the assumption that tumor patients might face delayed access to rehabilitation and the overall burden of cancer, which could limit their physical activity. To confirm the hypothesis, we selected a control group comprising healthy adults with similar characteristics, and a comparison group of traumatically amputated patients, as they appear to be the most similar to the tumor group in terms of patient characteristics. Interestingly, the results of our study did not support our hypothesis. Contrary to our expectations, patients who underwent amputations due to tumors demonstrated better performance in tests reflecting general daily mobility compared to those amputated due to trauma. Moreover, they also showed superior results in a battery of tests assessing high-activity levels (CHAMP), which, to our knowledge, was used for the first time on non-servicemember amputees.

Table 1 shows that, despite attempts to make the groups as similar as possible, the group of tumor amputees is, on average, about 20 years younger at time of assessment and 10 years younger at the time of amputation compared to the group of traumatic amputees. In contrast, the control group matched the tumor group concerning age. Time since amputation was with a mean of 22.4 years longer in the trauma group than in the tumor group with a mean of 12.3 years since amputation. If a time since amputation over 10 years may have an impact on the results is debatable. We found a correlation between age and test results for general daily mobility as well as for high-level activity. Consequently, age could be one reason for the differences in our study groups. From other studies we know that older amputees tend to have lower activity levels compared to younger ones [24, 25]. This is often due to a combination of factors such as decreased physical fitness, the presence of comorbidities, and a longer recovery time after amputation. Furthermore, literature mentions several other factors influencing physical activity in amputees like amputation level [26–29]. Interestingly, in both groups of tumor and trauma patients, participants with TF amputations performed better on the CHAMP compared to those with TT amputations. We expect this effect to be attributed to the small number of test subjects as we know that in TT amputees the knee joint and surrounding musculature can be used effectively for high-level mobility activities that require fast and explosive movements [30]. Furthermore, we know that TF amputees exhibit a significant amount of fatty degeneration in the quadriceps muscle, which likely influences gait and mobility [31]. Another reason for the differences in the study groups could be the cause of amputation can additionally influence the well-being of an individual post-amputation. Amputations resulting from traumatic events (e.g., accidents, combat injuries) can lead to significant psychological stress. The sudden and unexpected nature of the loss can make adjustment more challenging. Conversely, amputation as a treatment for cancer can bring a complex mix of emotions, such as fear of recurrence and coping with significant body image changes. The study by Torbjörnsson et al. found that some amputees perceived greater benefits, such as a reduced risk of death, compared to the costs of amputation. This perspective may particularly apply to tumor patients including relief from removing the disease [32].

Walking distance can be used as an assessment of overall mobility and quality of life in amputees [33]. According to a previous literature review, approximately 75% of individuals with a traumatic transtibial and 55% with transfemoral amputations can walk at least 500 m [34]. In our tumor cohort, only one patient reported a walking distance of less than 500 m (50 m) due to a postoperative nerve lesion. In the trauma group, two patients reported walking distances under 500 m (400 m and 200 m). Both patients were unemployed, whereas all patients in the tumor cohort who provided information about their walking distance were employed. Studies have shown that the percentage of amputees able to walk 500 m is higher among those who return to employment compared to those who do not [35, 36]. Questionnaires and functional tests, including the LCI, LCI-HD, TUG, 2MWT and 10MWT provide a comprehensive assessment of physical outcome and the current status as part of our clinical gait analysis [37, 38]. Comparison of mobility questionnaires, such as LCI and LCI-HD, and the performance-based tests showed better results for tumor amputees compared to trauma amputees with significant results for LCI, LCI-HD and 10MWT. With advances in surgery, prosthetic technology, and rehabilitation, amputees are increasingly able to return to or start with high-level activity. Consequently, there is a need for a high-level mobility outcome measure to assess progress during and after rehabilitation. The CHAMP, developed and tested by Gailey et al., was found to be a safe and reliable measure of high-level mobility in amputated servicemembers returning to duty [9, 17]. Our results indicate that, similar to general activity, scores for high-level activities measured by the CHAMP in trauma amputees are lower than those of tumor amputees. Compared to the healthy control group we have significantly lower scores in amputees. In all three groups, the CHAMP was significantly associated with the validated tests for general activity, similar to findings by Gailey at al., who demonstrated a positive relationship with the 6MWT and 2MWT [17, 39]. In comparison to servicemembers (SMs) studied by Gailey et al., our healthy cohort’s scores are almost comparable, suggesting the high activity level of the veterans. The highest amputees’ score in our study was 21 on the CHAMP, achieved by a TF tumor amputee, and 15 points by a TF trauma amputee. In contrast, Gailey at al. reported that two transtibial amputees scored 34 and 35 on the CHAMP [23]. The findings by Galey et al. [23] show that some SMs with lower limb amputation were able to achieve CHAMP scores comparable to their nondisabled SM peers. Interestingly, in both groups of tumor and trauma patients, participants with TF amputations performed better on the CHAMP compared to those with TT amputations. On the other hand, the group classified as “others”, where special surgical techniques are employed to imitate joint function, showed the best CHAMP results (maximum 17 points). All CHAMP items require a complex combination of muscular recruitment, movement strategies, prosthetic competency, and athletic abilities. These skills include balancing over the prosthesis; generating lower-limb power to produce fast, explosive movement; and producing efficient motion to start, stop and change directions. Regarding the relatively low scores in the CHAMP reached by the amputees, we would conclude that the participation in rehabilitation and special exercise programs afterwards can enhance subsequent activity levels. Our questionnaires revealed that of the 11 tumor amputees, 7 participated in some form of rehabilitation, physiotherapy, or gait training after amputation. In the trauma cohort, only half reported engaging in rehabilitation, physiotherapy, or gait training. The reasons for this discrepancy are not known. Interestingly, there was no correlation between CHAMP scores and participation in rehabilitation. The CHAMP, as a measure for high-level activity, is designed to be clinically feasible, even for amputees without any history of sports participation, requiring less than 15 min for administration and minimal equipment. In our study, all participants were able to complete the tasks with acceptable effort, as measured by BORG scale (Fig. 4).

Literature shows evidence that the relationship between motivation and participation is not direct, but influenced by facilitators, barriers, and processes to be active [40]. In our study it seems that tumor patients have stronger motivation concerning health issues than trauma patients (Table 5). There is ongoing debate regarding which characteristics play a more significant role in patients’ psychological adjustment and their quality of life: the disease leading to the amputation [41], age [42], gender [43], inability to walk [44] or reaching recommended levels of physical activity [45].

The limitations of this study include the relatively small number of patients, due to the extreme rarity of sarcomas and lower extremity amputations following these tumors. The relatively small sample size did not allow us to perform an age-matched control analysis within the two groups of limb amputation. Such an analysis could have excluded age as a potentially significant influencing factor, allowing for a closer examination of the effects of the level of amputation and the reason for amputation on physical outcome. Another limitation of our study is the selection of control participants. The group was matched visually on basic demographic variables including age, sex, height, and weight, rather than through formal statistical approaches such as propensity score matching. Due to the limited sample size, implementing advanced matching techniques was not feasible. We acknowledge that the use of formal matching methods would enhance methodological rigor and reduce selection bias in larger cohorts. Furthermore, the results are not applicable to the broader population of patients with amputations as we excluded amputations which are attributed to vascular diseases, particularly diabetes and peripheral artery disease. This group accounts for at least 70% of the amputations in Europe. Because of multiple comorbidities it is to expect that this group is less active. Moreover, factors, such as comfort, appearance, weight and usefulness are crucial regarding the use of a prosthesis in an amputee patient but were not directly investigated in this cohort [46, 47]. Bekrater-Bodmann et al. identified several factors influencing the embodiment of prosthesis in patients with lower limb amputation [48]. Prosthesis embodiment refers to the extent to which amputees perceive their prosthesis as a natural part of their body. As a conclusion the study underscores the importance of factors like sensory feedback, intuitive control, cognitive integration, and emotional well-being in fostering prosthesis embodiment. Enhancing these aspects can lead to higher prosthetic satisfaction and better overall and physical outcomes for patients with lower limb amputation. A potential bias in our study arises from the monocentric approach to patient selection. All patients were recruited from a single center, which leads to limited geographical and possibly demographic diversity.

Future studies should consider a multicentric approach to include a broader and more diverse patient population. This would help enhance the generalizability of the results and improve the applicability of the findings to various clinical and geographical contexts. Furthermore, studies involving a larger patient cohort could help verify the information gathered and investigate more closely their underlying causes. It would be interesting to assess how chemotherapy or tumor recurrences impact patients’ physical activity levels. Additionally, the type of prostheses used by patients may play a role in evaluating physical activity, especially in achieving high-level activities. In light of the limitations identified in our study, we strongly advocate for the establishment and utilization of comprehensive patient registries in the field of amputee care. Patient registries can provide valuable insights into the long-term outcomes and effectiveness of different treatment approaches, including prosthetic care.

## Conclusions

Rehabilitation factors such as lower limb strength, dynamic balance, and coordination are significantly associated with the CHAMP score and can be improved through appropriate measures. The CHAMP, designed as an assessment tool for high-level mobility in participants following major lower limb amputation in veterans, was successfully applied to a general amputee population. These results could serve as a guide for rehabilitation and aid in the development of suitable interventions to maximize high-level mobility capabilities, to uncover current status and deficits, and to motivate amputee patients by showing what is possible after an amputation. The study results support the CHAMP as a valid performance-based assessment tool for high-level mobility after lower extremity amputation.

## Supplementary Information

Below is the link to the electronic supplementary material.

Supplementary Material 1

## Data Availability

No datasets were generated or analysed during the current study.
