# Supplementary material for: High-level activity and access to sport following lower limb amputation due to malignant musculoskeletal tumors versus trauma: a prospective comparative study
Source: Arch Orthop Trauma Surg. 2025 Aug 21;145(1):418. doi: 10.1007/s00402-025-06037-x (PMC12370868; doi:10.1007/s00402-025-06037-x)
Supplement: Supplementary file 1 — Supplementary Material 1 [file 402_2025_6037_MOESM1_ESM.pdf]

Name: Datum:

Fragebogen zur sportlichen Aktivität und Lebensqualität nach Major Amputation der unteren Extremität

Bevor Sie den Fragebogen ausfüllen, möchten wir den Begriff Sport für diese Studie definieren:

Wir definieren Sport als Aktivität, die mit körperlicher Anstrengung (mit oder ohne Spiel und Wettkampf-Charakter) verbunden ist und mindestens eine halbe Stunde andauert, bei der körperliche Fähigkeiten vorausgesetzt werden oder erlernt bzw. verbessert werden.

1) Haben Sie vor Ihrer Amputation Sport getrieben?

- o Nein
- o Ja, regelmäßig — Trainingseinheiten pro Woche ______ / Zeit pro Trainingseinheit __________
- o Ja, unregelmäßig — Trainingseinheiten pro Woche ______ / Zeit pro Trainingseinheit __________

2) Wenn ja, welche Sportarten haben Sie vor der Amputation betrieben?

____

____

____

3) Wie lange vor der Amputation konnten Sie keinen Sport mehr machen?

____

4) Treiben Sie seit Ihrer Amputation Sport?

- o Nein
- o Ja, regelmäßig — Trainingseinheiten pro Woche ______ / Zeit pro Trainingseinheit __________
- o Ja, unregelmäßig — Trainingseinheiten pro Woche ______ / Zeit pro Trainingseinheit __________

5) Wenn ja, welche Sportarten haben Sie die letzten 6 Monate betrieben?

____

____

____

6) Wie stark haben sie folgende Aspekte motiviert mit Sport anzufangen?

	Gar nicht	etwas	stark	sehr stark
• Gut für die Gesundheit	☐	☐	☐	☐
• Ich befolge den Rat meines Arztes	☐	☐	☐	☐
• Unterstützung von Familie/Bekannten	☐	☐	☐	☐
• Erfahrung im Umgang mit der Prothese	☐	☐	☐	☐
• Bessere Beweglichkeit im Alltag	☐	☐	☐	☐
• Ich möchte meinen Körper formen	☐	☐	☐	☐
• Ich möchte mich mit anderen messen	☐	☐	☐	☐
• Ich möchte meine Grenzen austesten	☐	☐	☐	☐
• Ich habe soziale Kontakte durch den Sport	☐	☐	☐	☐
• Es gibt mir ein gutes Gefühl	☐	☐	☐	☐
• Es hilft mir abzuschalten	☐	☐	☐	☐
• Ich muss mitmachen: Wer schickt sie?:_________	☐	☐	☐	☐
• Sonstige, ________________________________	☐	☐	☐	☐

7) Wie stark motivieren sie folgende Aspekte weiter Sport zu treiben?

	Gar nicht	etwas	stark	sehr stark
• Gut für die Gesundheit	☐	☐	☐	☐
• Ich befolge den Rat meines Arztes	☐	☐	☐	☐
• Unterstützung von Familie/Bekannten	☐	☐	☐	☐
• Erfahrung im Umgang mit der Prothese	☐	☐	☐	☐
• Bessere Beweglichkeit im Alltag	☐	☐	☐	☐
• Ich möchte meinen Körper formen	☐	☐	☐	☐
• Ich möchte mich mit anderen messen	☐	☐	☐	☐
• Ich möchte meine Grenzen austesten	☐	☐	☐	☐
• Ich habe soziale Kontakte durch den Sport	☐	☐	☐	☐
• Es gibt mir ein gutes Gefühl	☐	☐	☐	☐
• Es hilft mir abzuschalten	☐	☐	☐	☐
• Ich muss mitmachen: Wer schickt sie?:_________	☐	☐	☐	☐
• Sonstige, ________________________________	☐	☐	☐	☐

8) Wie sehr hindern Sie folgende Aspekte daran Sport zu treiben?

	Gar nicht	etwas	stark	komplett
• Brauche ich nicht	□	□	□	□
• Kann ich nicht: Grund:____________________	□	□	□	□
• Keine Zeit	□	□	□	□
• Traue es mir nicht zu	□	□	□	□
• Fühle mich zu alt dafür	□	□	□	□
• Zu anstrengend	□	□	□	□
• Angst vor Verletzungen	□	□	□	□
• Blöde Fragen/ Blicke	□	□	□	□
• Angst vor Zurückweisung	□	□	□	□
• Ich bin immer auf Hilfe angewiesen	□	□	□	□
• Keine Transportmöglichkeit	□	□	□	□
• Ich weiß nicht wo ich Sport machen kann	□	□	□	□
• Kosten (Prothese/Beiträge/Transportkosten)	□	□	□	□
• Prothese fühlt sich fremd an	□	□	□	□
• Prothese sitzt nicht richtig	□	□	□	□
• Probleme mit dem Stumpf	□	□	□	□
• Schmerzen (Areal(e):__________)	□	□	□	□
• Sonstige, ______________________	□	□	□	□

9) Waren Sie vor Ihrer Amputation in Physiotherapie/Reha?

- ○ Nein
- ○ Ja (Wie lange, welche Zielsetzung?) ____________________________

10) Waren Sie nach Ihrer Amputation in Physiotherapie/Reha?

- ○ Nein
- ○ Ja (Wie lange, welche Zielsetzung?) ____________________________

11) Wie viel Zeit lag zwischen Amputation und Reha nach Ihrer Amputation?

______Tage

12) Besitzen Sie eines der genannten Hilfsmittel und wann wurde es jeweils verordnet? (Mehrfachnennung möglich)

	Nein	Ja	Anzahl	Erstverordnung	aktuelle Verordnung
• Eine Alltagsprothese	□	□	___	… …/… …/… …	… …/… …/… …
• Einen Alltagsrollstuhl	□	□	___	… …/… …/… …	… …/… …/… …

• Gehstütze(n)	□	□	___	... .../... .../... ...	... .../... .../... ...
• Eine Sportprothese	□	□	___	... .../... .../... ...	... .../... .../... ...
• Einen Sportrollstuhl	□	□	___	... .../... .../... ...	... .../... .../... ...
• Eine Badeprothese	□	□	___	... .../... .../... ...	... .../... .../... ...
• Sonstige, _____________	□	□	___	... .../... .../... ...	... .../... .../... ...

13) Nutzen sie während Ihrer sportlichen Aktivität ein Hilfsmittel?

- ○ Nein
- ○ Ja, weilche:____

14) Auf welchem Level betreiben Sie Sport? (Mehrfachnennung möglich)

- ○ Breitensport (alleine oder in der Gruppe ohne Rundenspielbetrieb)
- ○ Wettkampf
- ○ Leistungssport

15)Sind Sie Mitglied in einem Sportverein oder Fitnessstudio?

- ○ Nein
- ○ Ja, Fitnessstudio
- ○ Ja, Sportverein(e): ____

Falls ja, was waren ihre bisherigen sportlichen Erfolge?

____

16)

a. Hatten Sie Beschwerden oder Verletzungen aufgrund von Sport in den letzten 6 Monaten?

- ○ Nein
- ○ Ja

b. Wenn ja, welche Beschwerden/Verletzungen waren das?

____

____

c. Wenn ja, haben Sie die Beschwerden/Verletzungen von weiterem Sport abgehalten?

- ○ Nein
- ○ Ja, für _________Tage/ für _______Wochen / dauerhaft □

17) Finden Sie, dass Sie genug über Sportmöglichkeiten für Amputierte wissen?

- Nein,weil ____

- Ja, durch:
- Internet
- Zeitung
- Über andere Amputierte
- Spezialisierter Arzt (Chirurg, Reha, etc.)
- Hausarzt
- Physiotherapie
- Orthopädietechniker
- Sonstige, ____

18) Was müsste ihrer Meinung nach verbessert werden, damit mehr Patienten mit Amputation Sport treiben?

____

____

19) Welchen Tipp würden sie anderen Patienten mit Amputation gerne mitgeben um mehr Sport zu treiben?

____

Anhang A
Spezielle Tumorfragen

1) Wie sehr schränken sie folgende Tumorbeschwerden in ihrer sportlichen Aktivität ein?

	Habe ich nicht	Gar nicht	etwas	stark	sehr stark
• Fatigue (Müdigkeit)	□	□	□	□	□
• Schmerzen	□	□	□	□	□
• Durchfall	□	□	□	□	□
• Anämie (Blutarmut)	□	□	□	□	□
• Leistungsminderun	□	□	□	□	□
• Schwindel/ Gleichgewichtsstörung	□	□	□	□	□

2) Wie sehr schränken sie folgende Chemotherapienebenwirkungen immer noch in ihrer sportlichen Aktivität ein?

	Habe ich nicht	Gar nicht	etwas	stark	sehr stark
• Übelkeit	□	□	□	□	□
• Schmerzen	□	□	□	□	□
• Polyneuropathie (Gefühlsstörungen)	□	□	□	□	□
• Sehstörungen	□	□	□	□	□
• Durchfall	□	□	□	□	□
• Anämie (Blutarmut)	□	□	□	□	□
• Leistungsminderung	□	□	□	□	□
• Schwindel/Gleichgewichtsstörung	□	□	□	□	□

3) Wie lange nach Chemo waren Sie jeweils so beeinträchtigt, dass Sie keinen Sport treiben konnten?

___________ Tage

Anhang B
Spezielle Traumafragen

1) Wie sehr schränken Sie folgende Begleitverletzungen/Ausfälle in ihrer sportlichen Aktivität ein?

	Habe ich nicht	Gar nicht	etwas	stark	sehr stark
• Hand/Hände:	☐	☐	☐	☐	☐
• Arm/Arme:	☐	☐	☐	☐	☐
• Anderes Bein (inklusive Hüfte):	☐	☐	☐	☐	☐
• Wirbelsäule	☐	☐	☐	☐	☐
• Augen	☐	☐	☐	☐	☐
• Schwindel/Gleichgewichtsstörung	☐	☐	☐	☐	☐
• Nervenverletzung (Gefühlsstörungen)	☐	☐	☐	☐	☐
• Lähmung	☐	☐	☐	☐	☐
• Schmerzen	☐	☐	☐	☐	☐
• Hauttransplantation/Narben	☐	☐	☐	☐	☐
• Weichteildefekte	☐	☐	☐	☐	☐
• Ödeme	☐	☐	☐	☐	☐

2) Wie lange waren sie nach dem Trauma so beeinträchtigt, dass sie keinen Sport treiben konnten?

________ Tage

Anhang C

Allgemeine Gesundheitsfragen

20) Bestehen neben der Amputation noch weitere die Sportliche Aktivität beeinträchtigende Nebendiagnosen:

- Nein
- Ja

Falls ja:

- Muskelschwäche (Lähmung, Muskelerkrankung): Areal(e):___________
- Eingeschränkte Wahrnehmung: Areal(e):____________
- Rheuma
- Kardiovaskuläre Erkrankungen (Bluthochdruck, Herzinfarkt): _______________________
- Gefäßerkrankung (CVI/pAVK)
- Diabetes
- Arthrose anderer Gelenke: ____________
- Bronchitis /Asthma /COPD
- Nierenerkrankungen
- Schmerzen, Areal(e): ______________
- Phantomschmerzen
- Wunden am Stumpf
- Sonstige, ____

21)Haben Sie geraucht/rauchen Sie?

- Ja (wie viel und wie lange) ______Zigaretten am Tag, ______Jahre
- Nein

Danke für die Teilnahme!
